# Development of Condition Assessment Index of Ballast Track Using Ground-Penetrating Radar (GPR)

**DOI:** 10.3390/s21206875

**Published:** 2021-10-16

**Authors:** Fiseha Nega Birhane, Yeong Tae Choi, Sung Jin Lee

**Affiliations:** 1 Department of Transportation System Engineering, KRRI School, University of Science & Technology, 217, Gajeong-ro, Yuseong-gu, Daejeon 34113, Korea; fish@krri.re.kr; 2 Korea Railroad Research Institute, 176, Cheoldo-bangmulgwan-ro, Uiwang 16105, Korea; geolsj@krri.re.kr

**Keywords:** track inspection, ballast fouling, ballast thickness, dielectric permittivity, signal strength, time and frequency domains, ballast condition scoring index (BCSI), KRRI, GSSI

## Abstract

The condition of the ballast is a critical factor affecting the riding quality and the performance of a track. Fouled ballast can accelerate track irregularities, which results in frequent ballast maintenance requirements. Severe fouling of the ballast can lead to track instability, an uncomfortable ride and, in the worst case, a derailment. In this regard, maintenance engineers perform routine track inspections to assess current and future ballast conditions. GPR has been used to assess the thickness and fouling levels of ballast. However, there are no potent procedures or specifications with which to determine the level of fouling. This research aims to develop a GPR analysis method capable of evaluating ballast fouling levels. Four ballast boxes were constructed with various levels of fouling. GPR testing was conducted using a GSSI (Geophysical Survey Systems, Inc.) device (400, 900, 1600 MHz), and a KRRI (Korea Railroad Research Institute) GPR device (500 MHz), which was developed for ballast tracks. The dielectric permittivity, scattering of the depth (thickness) values, signal strength at the ballast boundary, and area of the frequency spectrum were compared against the fouling level. The results show that as the fouling level increases, the former two variables increase while the latter two decrease. On the basis of these observations, a new integrated parameter, called a ballast condition scoring index (BCSI), is suggested. The BCSI was verified using field data. The results show that the BCSI has a strong correlation with the fouling level of the ballast and can be used as a fouling-level-indicating parameter.

## 1. Introduction

The ballast layer plays an important role in keeping the sleepers in the correct position, in supporting repeated train loads, and in transferring the train load to the substructures. It also provides free drainage of water and prevents the growth of vegetation. Sound ballast materials should be strong, stable, and drainable. Railway ballast is subjected to both traffic loads and environmental changes and, as the ballast bed deforms and degrades, it adversely affects the performance of the railway track [1,2,3,4,5].

Ballasts become fouled because of the fragmentation of the ballast materials, the surface infiltration of weathered particles and coal droplets, and the upward migration of fines from the subgrade and sleeper wear [2,3,4,6]. When fouling becomes severe, excess pore water pressure is generated under cyclic loading that, in turn, degrades the track resiliency and stability in undrained conditions [2,4].

As a result, routine track inspections to access the ballast condition are an important aspect of track operations. Cost-effective tracks are needed as the demand for faster and heavier trains increases. To meet these needs, it is essential to improve the monitoring methods and the assessment of the track performance [1].

Railway track condition assessments mainly consist of monitoring the geometric parameters of the track using inspection vehicles. The geometric parameters are related to the positioning and wearing of the rail [7]. Track geometry deformation (i.e., track settlement) is the final phenomenon, arising because of various causes, such as track stiffness differences and subgrade defects, among others [1,8]. Nondestructive tests are widely used to assess the condition of the ballast and subgrade. Common nondestructive methods are visual inspections, the light-falling weight deflection method (LFWD), and electromagnetic methods (e.g., GPR) [1,2]. A lightweight deflectometer (LWD) involves dropping a known weight from a specified height onto a circular plate over soil and measuring the vertical surface deflection and the impact load. The LWD can also be used to determine the stiffness of the track [7,9,10].

Ground penetrating radar (GPR) is currently used more often to assess track ballast conditions. GPR uses electromagnetic signals to detect subsurface features, into which it transmits the electromagnetic pulses into the ground and measures the reflected signals. The changes in the dielectric properties of different materials and, hence, the layers, alter the electromagnetic wave propagation. Such a feature, along with its ability to acquire high-speed data without disturbing train operations, makes GPR a desirable inspection tool [2,5,6,8,11,12]. Nevertheless, GPR is not yet used in a systematic manner for railway track characterization or rehabilitation planning [8], one of the reasons being the lack of a simple and reliable method. With regard to this concern, numerous studies have been carried out. This study has been conducted with the aim of resolving this issue as well.

Conventional GPR analysis primarily involves the interpretation of the signal in the time domain, and is generally performed in specific locations or for a particular purpose, such as subgrade characterization or fouling detection [2,8,13]. Interpreting radar profiles is still a challenging and time-consuming task that, therefore, requires significant experience by GPR experts. Furthermore, there are no potent procedures to find the level and type of fouling quantitatively. Recently, many studies have focused on identifying the thicknesses and fouling levels of railway ballast by means of a frequency-domain analysis, in addition to a time-domain analysis [2,8,11,13,14,15,16,17].

Fontul et al. [8] attempted to identify infrastructure changes by adopting short- and long-sliding windows. For these sliding windows, they calculated the area under the signal in the time and frequency domains. The peaks of these areas, and the peaks of the differences of the areas between the short and long sliding windows, were utilized to detect anomalies in ballast tracks.

Anbazhagan et al. [16], and Alani et al. [18], showed that as the fouling level increases, the electromagnetic wave velocity decreases and the dielectric constant increases. De Chiara [19] points out that the presence of moisture can significantly increase the dielectric constant of a medium. In addition, they showed that the dielectric permittivity can vary, not only according to the frequency of the GPR, but also according to the antenna configuration of the device.

Roberts et al. [20] adopt a void-scattering amplitude envelope approach to assess the condition of the ballast. After comparing the energy between the clean and fouled sections, they showed that the loss of energy is higher in fouled ballast, as it has fewer void spaces.

Most GPR applications achieve the detection and localization of buried features and discontinuities and/or the estimation of the electromagnetic properties of materials by performing signal processing tasks in the time domain. However, time-domain methods do not account for the frequency-dispersive properties of media, and do not consider the signal phase [8]. In the frequency domain, it is also easy to filter out electromagnetic interference.

Various attempts have been made to detect the fouling level of ballast using a frequency spectrum analysis, in which the time-domain GPR data are converted to the frequency domain using the Fourier transform [8,13,21]. Silvast et al. [21] showed that the area of a signal in the frequency domain is higher in clean ballast compared to fouled ballast. Shao et al. [13] used a 800 MHz antennae to evaluate the track at the network level. They proposed an automatic ballast condition assessment method using the maximum peak in the frequency domain.

Birhane et al. [11] developed a 500 MHz GPR device (referred to as a KRRI GPR device). The device has eight channels and can eliminate sleeper noise at the GPR surveying stage. In addition, an algorithm that automatically detects the thickness of the ballast layer was developed. It was found that the caliber of the thickness detection algorithm differed in the clean and fouled ballast sections of the track.

In summary, although various studies have found correlations between GPR parameters, such as the dielectric permittivity and the area of the frequency spectrum and the fouling level, there are no potent procedures by which to ascertain the level of fouling. The determination of the level and type of fouling of the ballast layer from GPR analysis remains challenging.

This paper aims to assess the fouling level of the ballast using both time- and frequency-domain parameters. The correlation of the fouling level of the ballast with the time- and frequency-domain parameters, such as the dielectric permittivity, signal strength at the bottom ballast boundary where materials change into reinforced trackbed or sublayer soil, scattering of the ballast thickness values, and the area of the frequency spectrum, is investigated thoroughly.

## 2. Methods and Tools for GPR Survey

### 2.1. Experimental Setup

Birhane et al. [11] pointed out that fouled ballast shows more signal scattering, especially at the boundary of the ballast and reinforced subgrade. In this study, to assess the fouling level of the ballast meticulously, further GPR surveys were conducted on lab-built and actual ballast tracks.

In the lab, the ballast tracks were simulated using four ballast boxes with distnict fouling levels. The four ballast boxes represent the stages of fouling on a ballast track: (1) clean (Box 1); (2) partially fouled (Box 2); (3) fully fouled (Box 3); and (4) Box 4, which has increasing fouling material content according to the depth, as illustrated in Table 1. The fouling material is added to the ballast aggregate in all cases at predetermined ratios and mixed before the boxes are filled.

**Figure 1:**
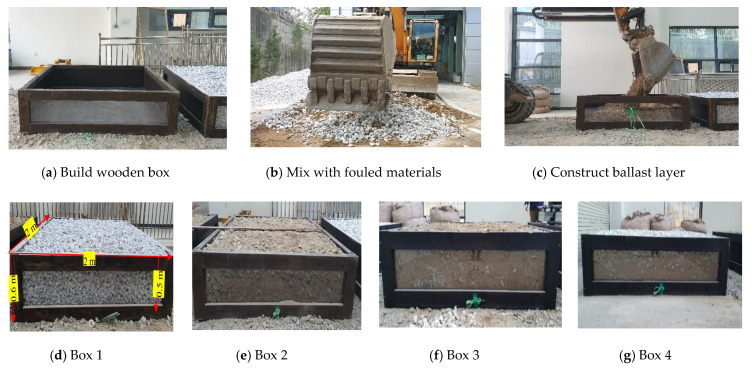
Construction of ballast boxes.

The ballast tracks consisted of 2 m × 2 m × 0.6 m wooden frames. The front side wall was composed of a transparent material to facilitate visual inspections. Figure 1 illustrates the construction procedure of the ballast boxes. Using an excavator, the gravel and fouling materials were mixed. This mixed material was then poured into the boxes, as shown in Figure 1.

**Figure 2:**
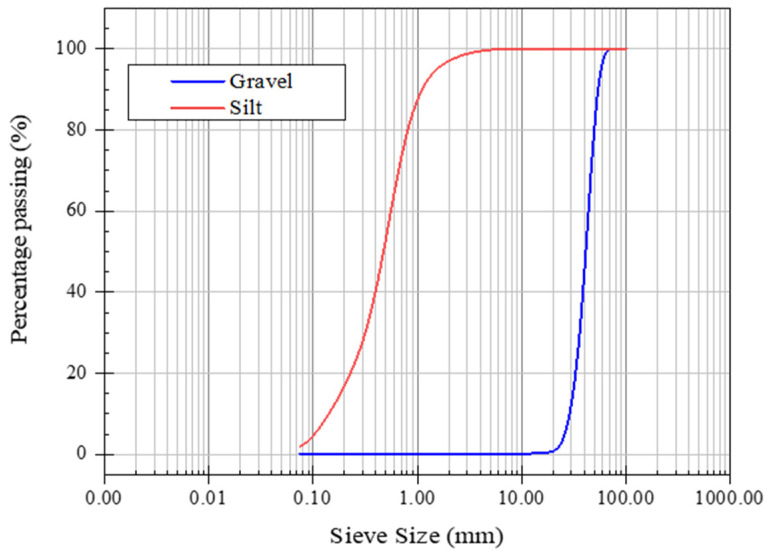
Grain size distribution of the ballast gravel and fouling silt.

Figure 2 shows the gradation of the ballast aggregates and the fouled materials. Only 0.2% of the ballast aggregates can pass through 4.75 mm and 0.075 mm sieves. In contrast, in the fouling silt material, 100% and 1.9% of the material can pass through the 4.75 mm and 0.075 mm sieves, respectively. The fouling index can be calculated by Equation (1), suggested by Selig and Waters [22]:

(1) $$FI=P_{\%}^{4}+P_{\%}^{200}$$

where $P_{\%}^{4}$ is the percentage by mass of the sampled ballast material finer than the 4.75 mm (No. 4) sieve, and $P_{\%}^{200}$ is the percentage by mass finer than the 0.075 mm (No. 200) sieve.

The corresponding fouling indices of each of the ballast boxes were 0.4, 34.23, 51.15, and 30.08 for Boxes 1 to 4, respectively, as shown in Table 1.

### 2.2. Surveying Equipment

A KRRI (Korea Railroad Research Institute, Uiwang-si, South Korea) GPR device with an operating frequency of 500 MHz, developed for ballast track surveys, and commercial GSSI (Geophysical Survey Systems, Inc., Nashua, NH, USA) devices, with operating frequencies of 400, 900, and 1600 MHz, were used for laboratory tests. The KRRI GPR device was developed for field surveys, especially of ballast tracks. Accordingly, the KRRI GPR device was used for verification tests on the Gyeongbu high-speed railway [11].

**Figure 3:**
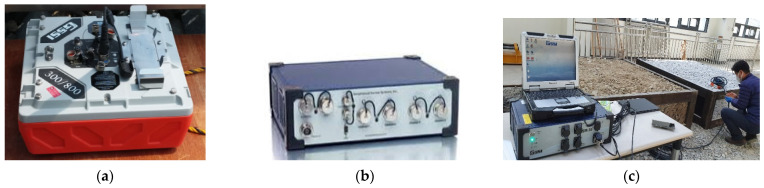
GSSI GPR device: (**a**) GSSI antenna; (**b**) GSSI SIR-30 controller; and (**c**) GSSI ballast survey.

**Figure 4:**
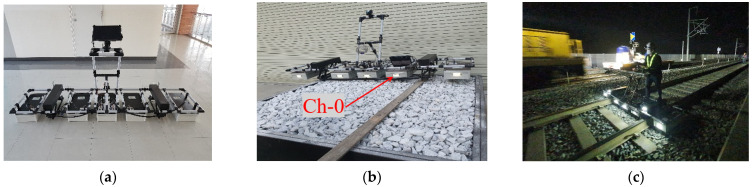
(**a**) KRRI GPR device; (**b**) KRRI ballast survey in the lab; and (**c**) KRRI ballast survey on the Gyeongbu HSR line.

In order to avoid the boundary effects of the ballast boxes, the centerlines of the boxes were surveyed. The test setup and operation using GSSI and KRRI devices are illustrated in Figure 3 and Figure 4, respectively. The KRRI device has eight channels, four on the left four on the right. Thus, in order to scan the centerline only one side antenna (channel-0) was used during the lab test (pleases see Figure 4b).

## 3. Development of the Ballast Condition Scoring Index

### 3.1. Preprocessing of Collected GPR Signals

The GPR dataset should undergo preprocessing procedures before further analysis is carried out because it is always possible for the GPR signals to have noise from the surrounding environment [11,12,23,24,25]. For the KRRI device, an in-house program was used [11], while Prism2 was used for the data processing with the GSSI devices. The general preprocessing procedures applied in this paper are explained below [11,23]:

(1) Frequency filtering (high-pass and low-pass filter) to remove higher and lower frequency noises.
  - High-pass filtering: the cutoff frequency is one-quarter (1/4) of the operating frequency
  - Low-pass filtering: the cutoff frequency in seven-fourths (7/4) of the operating frequency
(2) Time zero correction to remove the air ground interface (i.e., the gap between the GPR antenna and the surface of the medium)
(3) Gain: a linear gain function was applied to compensate for the natural attenuation of the signal
  - A linear gain function, with a slope ranging from 0.5 to 5%, can be used depending on the attenuation of the signal.

**Figure 5:**
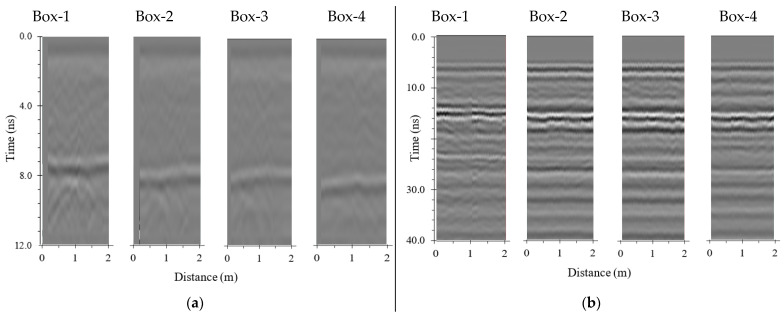
(**a**) B-scan 900 MHz GSSI device, and (**b**) B-scan 500 MHz KRRI device using Channel 0.

Figure 5 shows preprocessed B-scan images obtained from the GSSI and KRRI devices. As shown in the figure, it is difficult to discern the fouling level of the ballast from the B-scan image alone. Both the GSSI and the KRRI devices can detect the boundary between the ballast layer and the sublayer. The boundary in Figure 5 would be assumed to exist at the location where the GPR signal jumps. B-scan images can hardly deliver information other than the boundary.

In order to evaluate the ballast condition with GPR signals, the relevant parameters should be investigated. On the basis of the literature and experience with GPR, four parameters were selected: the dielectric permittivity; signal strength at the boundary; standard deviation (i.e., signal scattering); and area of the frequency spectrum. The standard deviation comes from the calculation of the scattering of thickness values generated by the ballast thickness detection algorithms suggested by Birhane et al. [11]. The area of the frequency spectrum can be calculated by converting the GPR signals into frequency domain images. On the basis of the investigation of each parameter, a new integrated parameter, called the ballast condition scoring index (BCSI), is suggested. The details are illustrated below.

### 3.2. Dielectric Permittivity Variation

The dielectric permittivity is a measure of the speed at which an electromagnetic signal passes through media. The dielectric permittivity can indicate the fouling level of the ballast by comparing the measured value with that of clean ballast. The dielectric permittivity constant (ξ) can be calculated at a known depth (or thickness) of the medium using Equation (2):

(2) $$\xi ={\left(\frac{C\times \Delta t}{2D}\right)}^{2}\;$$

where *C* is the speed of light 3 × 10^8^ m/s; Δt is the time it takes for the GPR signal to reach the boundary of the ballast (s); and D is the depth (thickness) of the ballast (m).

**Figure 6:**
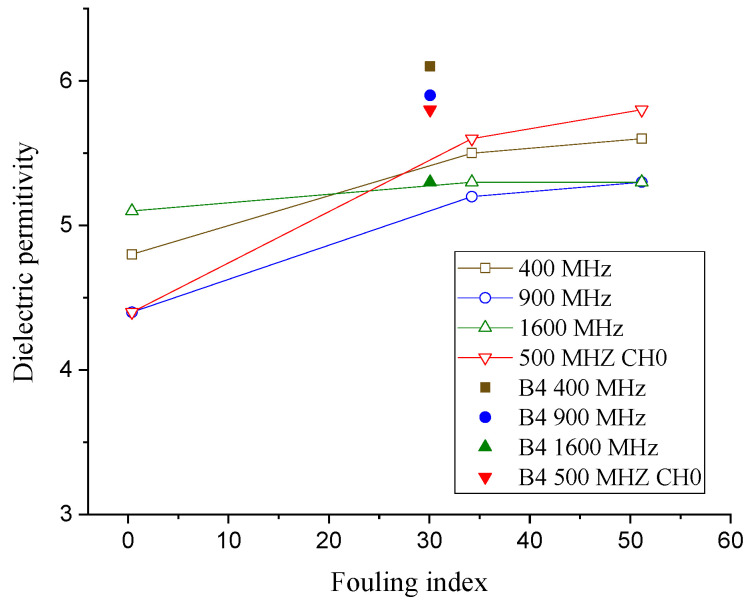
Dielectric permittivity vs. the fouling level at various frequencies.

Several studies have found that the dielectric permittivity increases as the fouling level increases [16,18,19,26]. Figure 6 shows that the dielectric permittivity calculated at the ballast boxes varies according to the fouling index. The dielectric permittivity of Box 4 is indicated with solid markers and is different from the others (hollow markers). As illustrated in Table 1, the fouling level of Box 4 increases as the depth increases, whereas the others remain constant within each layer. For this reason, Box 4 appears not to follow the trend according to the fouling index. Therefore, the results of Box 4 are included in the graphs but are not included in any further analysis in order to develop the ballast condition scoring index.

As anticipated, the more fouled ballast boxes have higher dielectric permittivity levels. The dielectric permittivity values ranged from 4.4 for Box 1 (clean), to 5.8 for Box 3 (fully fouled). The range of the dielectric permittivity of the ballast layers matches those from the literature [16,18,19,26,27]. Note that the dielectric permittivity of Box 4 with layered fouling is the highest, in all GPR frequencies. This observation indicates that multiple layers of materials could reduce the speed of the electromagnetic waves and raise the dielectric permittivity of the media.

In order to use the dielectric permittivity as a ballast-fouling-level-indicating parameter, the thickness of the ballast must be known beforehand. This limits the applications of the dielectric permittivity to assess the fouling level.

### 3.3. Boundary Estimation and Variation

**Figure 7:**
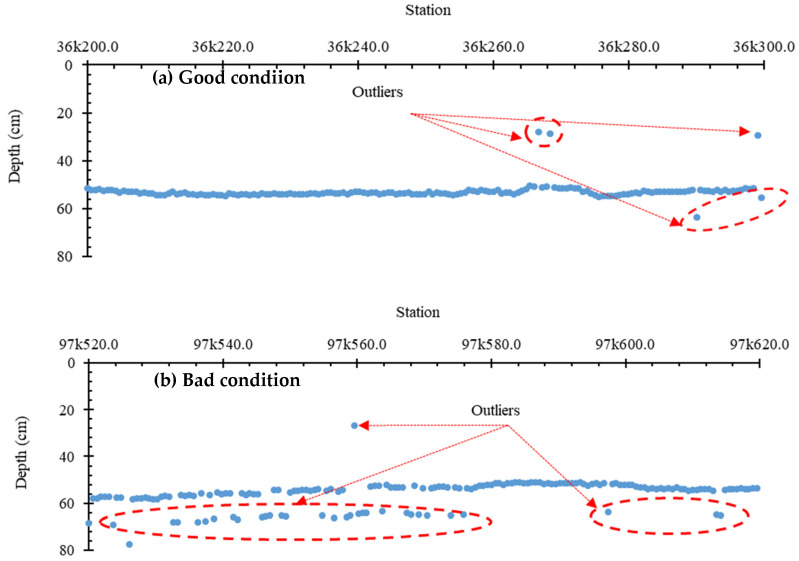
Ballast thickness detection (**a**) at a good site, and (**b**) at a poor site.

The automatic thickness detection algorithm, by Birhane et al. [11], showed contrasting results between the good condition and the fouled condition of ballast track. The good condition, i.e., ballast that was either clean or had little fouling, shows fairly continuous thicknesses, as shown in Figure 7a. However, the poor ballast condition, i.e., fouled ballast, resulted in scattered thickness detection according to the algorithm, as shown in Figure 7b.

This feature implies that the scattering of the thickness values might indicate the fouling level. Scattering, in this case, can be expressed quantitatively as the standard deviation of the ballast thickness. In this regard, the standard deviation of the depth (thickness) value generated by the depth detection algorithm (DVS) is selected as a GPR parameter.

As mentioned in Section 3.2, this stems from the fact that clean ballast is uniformly graded ballast, with more air voids and different physical and electromagnetic behaviors between the ballast and the subgrade, meaning that there are large differences in the dielectric constant. This makes the boundary more apparent for the thickness detection algorithm, whereas fouled ballast has finer particles filling the air voids and smaller aggregates segregating to the bottom section. This causes the electromagnetic characteristics of the ballast layer to resemble those of the subgrade. It is also important to reiterate that the presence of finer particles results in greater energy attenuation, that makes the boundary more obscure [20].

**Figure 8:**
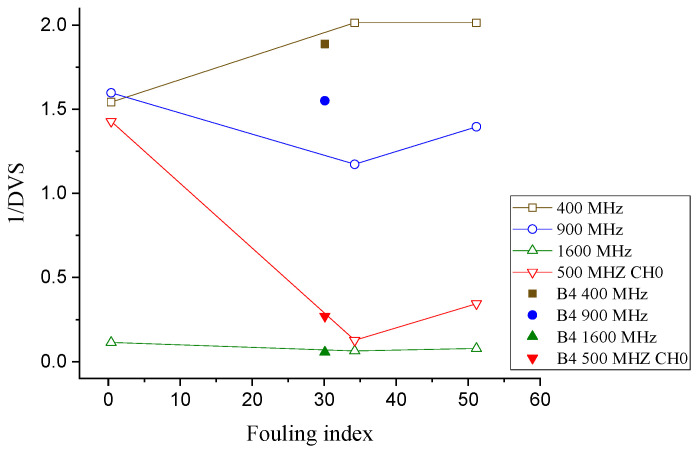
Inverse of the standard deviation of the thickness values generated by the according algorithm vs. the fouling level at various frequencies.

The standard deviation (signal scattering) of the thickness values generated by the depth (thickness) detection algorithm (DVS) was compared with the fouling level at various frequencies. As shown in Figure 8, except for 400 MHz, when the fouling level changes from a clean to a fouled condition, the inverse of the standard deviation of the depth (thickness) values from the algorithm (1/DVS) decreases. However, when the fouling index rises from 34.23 to 51.15 (Box 2 to Box 3), 1/DVS increases instead of decreasing.

Although the inverse of DVS seems to contradict the anticipated trend, when the fouling level rises above a certain level, it is significant for distinguishing a good condition ballast from a fouled and/or semi-fouled ballast. This was verified by the analysis of the results of the GPR scan on the Gyeongbu HSR line; details are presented in Section 3.6.

### 3.4. Signal Strength

The signal strength at the boundary of the ballast is another GPR parameter that can indicate the fouling level of the ballast layer. Fouled ballast materials (i.e., fine particles) tend to absorb electromagnetic waves and, thus, the reflected signals become weak. This phenomenon results in lower energy at the boundary [20]. Clean ballast is uniformly graded and contains more air voids and, therefore, shows a clearer difference between the clean ballast layer and the subgrade (here, the bottom of the box). The contrasting difference at the boundary gives rise to a large reflection. The large reflection results in a strong echo back to the GPR antenna [28].

**Figure 9:**
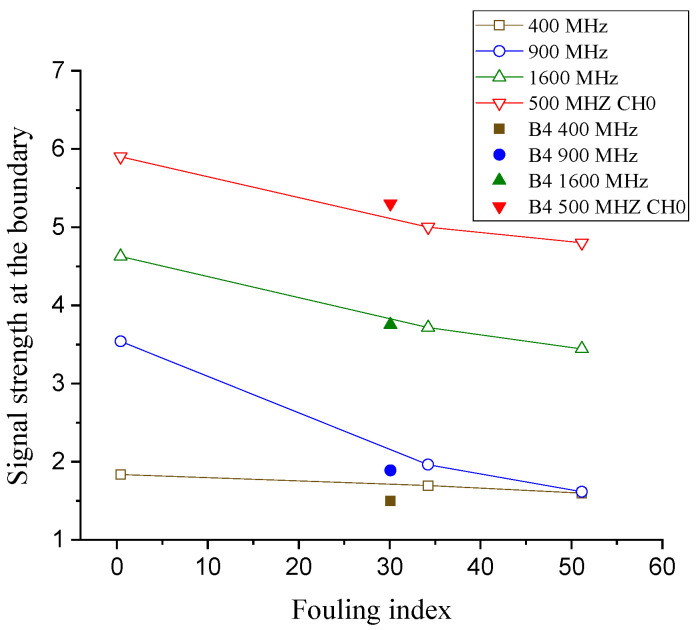
Signal strength at the boundary vs. fouling level with various frequencies.

The amplitudes of the signals at the ballast boundary are compared against the fouling level (see Figure 9). Because the amplitudes in both the time domain and frequency domain vary significantly due to different frequencies and devices, an adjustment should be applied for a reasonable comparison. The adjustment factors (refer to Table 2) are determined by the amplitude of the strongest signals of each frequency and are utilized to normalize the signal strength. The strength used in this section is the amplitude of the reflected signal at the boundary, where the KRRI algorithm calculates the boundary [11].

As shown in Figure 9, the more the ballast is fouled, the greater the energy loss and signal attenuation and, hence, the signal strength at the boundary is lower. As stated earlier, this is mainly caused by the loss of energy, which is higher in fouled ballast, as there are fewer void spaces [20].

### 3.5. Area of the Frequency Spectrum of the Signal

Silvast et al. [21] compared the area of a signal in the frequency domain along fouled and clean ballast sections and showed that the area is significantly larger in clean ballast. Therefore, the area bounded by the frequency spectrum is used as the frequency-domain analytic parameter to assess the fouling level.

In this study, the average of the area bounded by the frequency spectrum of the signal is calculated for each box and compared with regard to the fouling level. Similar to the signal strength, the adjustments have been made for the calculated areas, as shown in Table 3.

**Figure 10:**
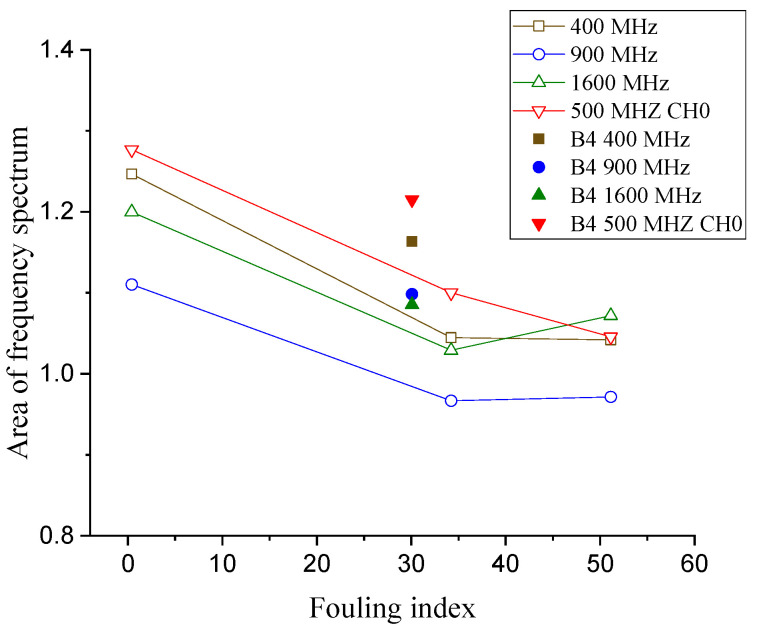
Area of the signal under the frequency domain vs. the fouling level at various frequencies.

As shown in Figure 10, the more the ballast is fouled, the smaller the area bounded by the frequency spectrum of a signal becomes. This is also in agreement with earlier reports in the literature [8,21].

### 3.6. Ballast Condition Scoring Index (BCSI) vs. the Fouling Level

After thoroughly analyzing the correlations among the four parameters above with the fouling level, a new integrated parameter called the ballast condition scoring index (BCSI) was suggested to indicate the fouling level. The BCSI is a function of the signal strength at the ballast boundary (SSb), the depth (thickness) value scattering (DVS), and the area of the frequency spectrum of the signal (A_fft_). It is defined as Equation (3).

(3) $$\mathrm{BCSI}\;={\left(\mathrm{SSb}+\frac{1}{\mathrm{DVS}}\;\right)}^{A_{\mathrm{fft}}}$$

To come up with Equation (2), various combinations of the three parameters, starting from direct superimposition and simple multiplication, were investigated and their performances were compared. Accordingly, the function in the form of Equation (2) gives the best result. The dielectric permittivity is excluded because the thickness of the ballast should be known beforehand in order to use the dielectric permittivity. The BCSI is a coherent parameter to indicate the fouling level of the ballast and is, theoretically, more accurate and stable for the fouling analysis, as it includes both the time-domain and the frequency-domain analyses. The GPR parameters, especially the ones related to the time-domain analysis, can be affected by a number of factors in addition to the fouling level, such as the thickness of the ballast layer, the moisture content, and the type of fouling material. For example, the signal strength can be affected by the thickness of the ballast, i.e., even if the condition of the ballast is identical, as the thickness of the ballast increases, the energy level at the bottom boundary will be lower because of the higher level of attenuation [29].

Furthermore, it is reported in the literature that the moisture level influence in a frequency-domain analysis is minor compared to that in a time-domain analysis [8]. Therefore, combining the time domain and the frequency domain into a single integrated parameter enables the stabilization of the parameter and uses their combined advantages to assess the condition of the ballast, which is tampered with by multiple variables.

**Figure 11:**
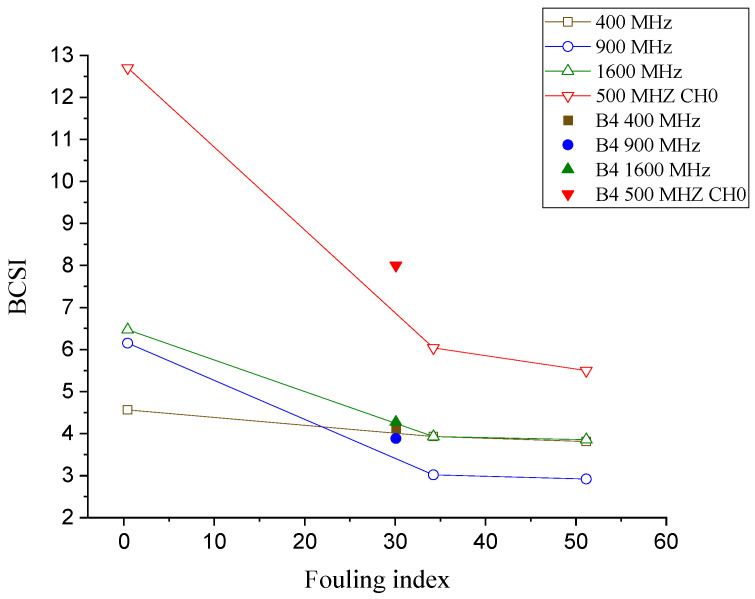
Ballast condition scoring index (BCSI) vs. the fouling level at various frequencies.

The correlation between the fouling level and the ballast condition scoring index (BCSI) is presented in Figure 11. A higher scoring index means a better ballast condition. Once more, all of the frequencies show a trend similar to those of the dielectric permittivity, the signal strength at the boundary, and the area of the frequency-domain graph.

The results show that the parameters can clearly distinguish between clean and fouled ballast. Clean ballast has low dielectric permittivity, high signal strength at the bottom boundary, low scattering of the thickness value, and a higher area of the frequency spectrum and, thus, results in a high scoring index.

The KRRI 500 MHz GPR device appears to discern fouling levels with the BCSI. For the GSSI device, 900 and 1600 MHz antennas seem to have the capability to figure out the fouling index; however, a 400 MHz frequency would not be appropriate to evaluate the fouling level of the ballast layer in operating lines. Birhane et al. [11] proved that, in actual tracks, the GPR data collected using commercial GPR devices shows significant noise around and under sleepers. The KRRI GPR device, on the other hand, which, unlike the commercial devices, has a transmitter and receiver antennae at separate boxes, can successfully eliminate the noise, especially at the lower section of the ballast layer. The arrangement of the antennae, and the magnetic sensors that detect fasteners on the sleepers, enable the KRRI device to scan the ballast in between the sleeper, thereby significantly minimizing the interference of the sleepers. In this sense, the KRRI GPR device is used for verification in operating lines.

## 4. Verification of the Ballast Condition Scoring Index on a High-Speed Railway

In order to apply the suggested index, BCSI, to the maintenance of operating lines, the BCSI needed to be verified through field testing. For this purpose, the Gyeongbu high-speed railway was selected, and more than 10 km of track was scanned using a KRRI GPR device at various sites.

**Figure 12:**
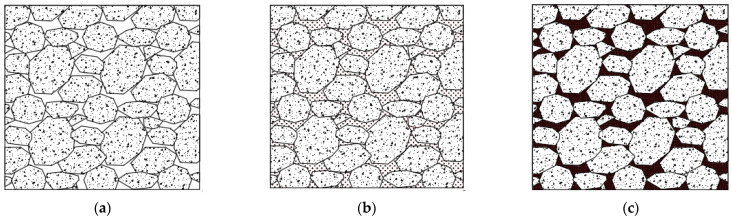
Fouling levels of ballast: (**a**) clean ballast; (**b**) partially fouled ballast; and (**c**) fully fouled ballast.

The test pits were excavated at randomly selected sections of bridges, tunnels, and embankments along the track. Because of the lack of an appropriate sampling technique, a quantitative fouling index could not be obtained on the Gyeongbu HSR line. Instead, the ballast condition was carefully assessed visually at selected excavation pits. After visually inspecting the excavated pits, the condition of the ballast was qualitatively graded according to the fouling level category suggested by Selig and Waters [22] (refer to Table 4 and Figure 12). On the basis of the description of the ballast condition, and the graphical description shown in Figure 12, the fouling index (FI) was estimated in a quantitative manner. Table 5 illustrates the major sites for the GPR survey, which were, in this case, the Banwall, Baebang, Pyeongtaek, and Gemeo sites. The details of the ballast thickness, a description of the fouling levels, and other factors pertaining to the test pits are summarized in Table 5.

**Figure 13:**
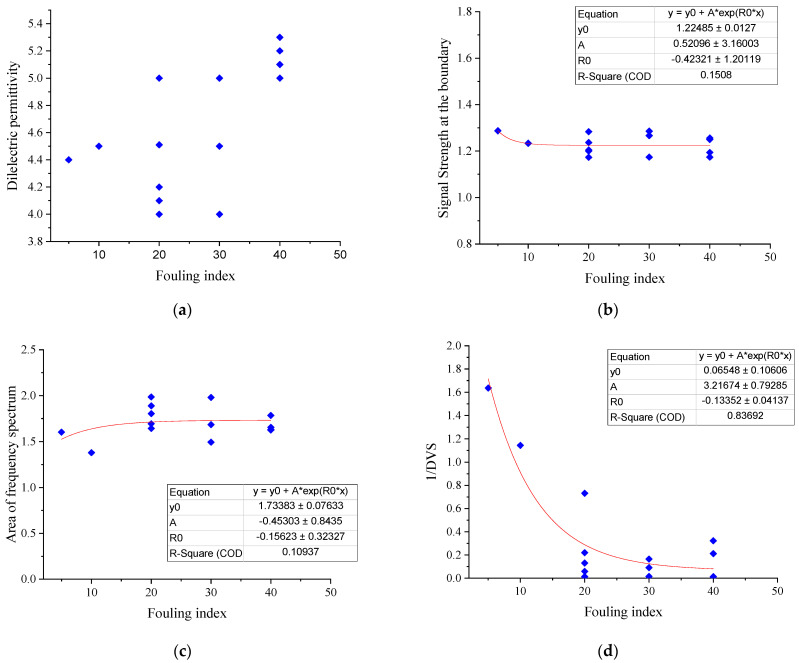
Relationships between the qualitative fouling level of the ballast and (**a**) the dielectric permittivity; (**b**) signal strength at the boundary; (**c**) area of the frequency spectrum; and (**d**) inverse of the standard deviation of the depth (thickness) values.

The relationships among the fouling levels of the ballast along the Gyeongbu HSR line and the dielectric permittivity, signal strength at the boundary, area of the frequency domain, and the standard deviation of the ballast thickness value are summarized in Figure 13. For all parameters, a 20m average length was taken so as to obtain the representative values of a certain location. As the fouling level of the ballast increases, the dielectric permittivity and the standard deviation of the thickness values from the algorithm increase, whereas the signal strength at the ballast boundary, and that of the area of the frequency spectrum, decrease.

The GPR parameters are closely correlated with the fouling level along the HSR line; however, the correlation is not as strong as those from ballast box tests. This occurs because the ballast boxes exist in a well-controlled environment, whereas on the actual track, the thickness of the ballast layer, the moisture content, the type of fouling materials, and the fouling level vary along operating lines.

**Figure 14:**
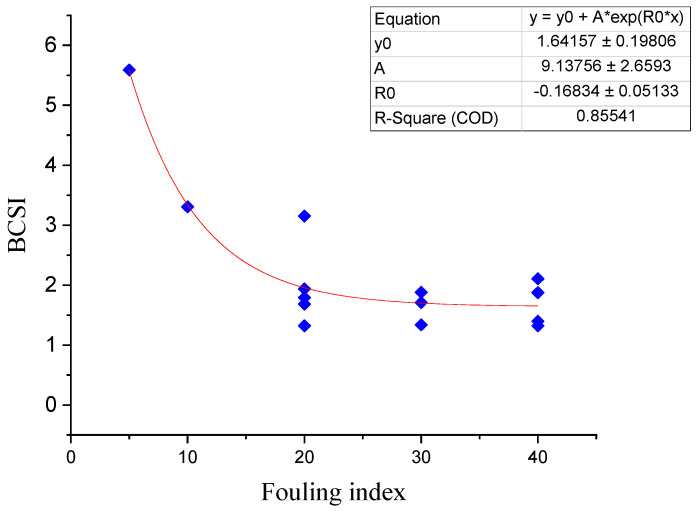
Relationship between the fouling level of the Gyeongbu HSR and the BCSI.

The ballast condition scoring index (BCSI) has a strong correlation with the fouling level, where a good ballast condition has a higher scoring index. A comparison of Figure 13 with Figure 14 shows the integrated parameters. The BCSI performs much better in indicating the fouling level of the ballast layer than the individual parameters. As shown in the figures, an exponential regression was performed on all the variables in Equation (2), including the BCSI. The coefficient of determination (R^2^) was 0.15, 0.11, 0.84, and 0.86 for the signal strength at the boundary, area of the frequency spectrum, the inverse of the standard deviation of the depth (thickness) values from the algorithm (1/DVS), and the ballast condition scoring index (BCSI), respectively.

The results, both in the lab-built boxes and the actual track, show that the correlation of the fouling level with the BCSI is not only stronger, but also more stable, than with the individual parameters. This demonstrates that the BCSI can be used as a tool to indicate the possible fouled section of the track, where detailed investigation is needed. It can also be expanded and/or improved by including more fouling level informative time-domain and frequency-domain parameters, which might be investigated in future works, i.e., the BCSI can be continuously updated to include additional parameters that can possibly indicate the fouling level of ballast. With further verification, and the possible inclusion of more fouling-level-indicating parameters, the BCSI can be refined into a more robust and reliable ballast-fouling assessment index. Given the ability of GPR devices to be easily mounted on track-inspection or commercial trains, the BCSI can also serve as a swift real-time ballast-condition-assessment and monitoring tool.

## 5. Conclusions

Track irregularities are the major parameters when determining the need for track maintenance. Fouling of the ballast is considered one of the most important factors causing track irregularities or the settlement of the ballast track. Many researchers have attempted to develop technologies to assess ballast conditions, and GPR has served as a suitable nondestructive tool.

This paper aims to develop an index and/or a method to evaluate the ballast condition in a practical and efficient manner. To do this, four ballast boxes were built with different fouling levels, and GPR signals were then collected. A GSSI GPR device with operating frequencies of 400, 900, and 1600 MHz, and a KRRI GPR device with an operating frequency of 500 MHz, were utilized to develop an index able to assess the ballast-fouling level.

The fouling levels of the ballast were compared with four parameters (dielectric permittivity, signal strength at the boundary, standard deviation (scattering) of the thickness, and the area of the frequency domain signal). The comparison shows that clean ballast has low dielectric permittivity, high signal strength at the bottom boundary, low scattering of the thickness value, and a larger area of the frequency-domain signal. On the basis of this observation, a new integrated parameter called the ballast condition scoring index (BCSI) is suggested.

The ballast condition scoring index (BCSI) can discern different fouling levels because it has a good correlation with the fouling level of an actual railway track line operating in Korea (the Gyeongbu high-speed railway). Accordingly, it can be used as a tool to indicate the possible fouled section of the track, where detailed investigation is needed. It can also be improved by including more fouling-level indicative, time-domain, and frequency-domain parameters.

Since GPR devices can easily be mounted on track-inspection or commercial trains, the BCSI can also serve as a swift real-time ballast condition assessment and monitoring tool.

## Figures and Tables

**Table 1:** Test Track Ballast Boxes with Various Fouling Levels.

	Box 1	Box 2	Box 3	Box 4
**Ratio of fouling material to clean ballast**	0	0.5	1.0	0–1.0
**Fouling index**	0.4	34.23	51.15	30.08
**Description**	Clean	Partially fouled	Fully fouled	Partially fouled (layer fouled) * 5th (0.00), 4th (0.25), 3rd (0.50), 2nd (0.75), 1st layer (1.00) *

* Bottom to upper layer (1st layer = bottom, 5th layer = surface layer), () = ratio of fouling material to clean ballast.

**Table 2:** Adjustment Factors to Normalize the Signal Strength.

Frequency	Adjustment Factor	Remark
400 MHz	15 × 10^3^	GSSI
900 MHz	3 × 10^3^	GSSI
1600 MHz	6 × 10^3^	GSSI
500 MHz	9 × 10^3^	KRRI

**Table 3:** Adjustment factors to normalize the area of the frequency domain.

Frequency	Adjustment Factor	Remarks
400 MHz	4 × 10^10^	GSSI
900 MHz	2 × 10^9^	GSSI
1600 MHz	2 × 10^10^	GSSI
500 MHz	4 × 10^9^	KRRI

**Table 4:** Fouling Index and Corresponding Ballast Condition Categories.

Fouling Index ^1^	Description of Ballast Condition
<1	Clean
1 to <10	Moderately clean
10 to <20	Moderately fouled (Partially fouled)
20 to <40	Fouled
>40	Highly fouled (Fully fouled)

^1^ Fouling index category [22].

**Table 5:** Condition of the Ballast Layer at Selected Trial Pits of the Gyeongbu HSR Line.

Site	Track	Location	Thickness (cm)	Description of the Fouling Level	Category According to Selig and Waters		Fouling Index (Fi)
Banwall	T1	36k260.0	53	Very good condition with slight fouling	Moderately clean	1 to <10	5
		36k294.0	48	Heavy fouling boundary at the bottom	Fouled	20 to <40	30
		36k397.0	53	Good to moderate fouling	Moderately clean	1 to <10	10
Baebang	T2	97k210.0	>36	Highly fouled—cannot be hand excavated beyond 36 cm.	Highly fouled	≥40	40
		97k530.0	>53	Moisture detected on lower section.	Highly fouled	≥40	40
Geomo	T1	241k295.0	56	Highly fouled—cannot be excavated beyond 36 cm.	Moderately fouled	10 to <20	20
		241k350.0	>54	Lower section has moisture.	Highly fouled	≥40	40
		241k370.0	56	Moderate fouling.	Fouled	20 to <40	30
		241k460.0	69	Serious fouling.	Moderately fouled	10 to <20	20
		241k530.0	>62	Cannot be hand excavated beyond 54 cm.	Highly fouled	≥40	40
	T2	241k347.0	60	Fouling moderate-serious. Moisture presence at the bottom section.	Moderately fouled	10 to <20	20
		241k370.0	53	Moderate fouling	Fouled	20 to <40	30
		241k405.0	>35	Serious fouling/not to bottom	Moderately fouled	10 to <20	20
		241k446.0	70	Moderate fouling	Moderately fouled	10 to <20	20
